# Prevention of thrombotic risk in hospitalized patients with COVID-19 and hemostasis monitoring

**DOI:** 10.1186/s13054-020-03000-7

**Published:** 2020-06-19

**Authors:** Sophie Susen, Charles Ambroise Tacquard, Alexandre Godon, Alexandre Mansour, Delphine Garrigue, Philippe Nguyen, Anne Godier, Sophie Testa, Jerrold H. Levy, Pierre Albaladejo, Yves Gruel, on behalf of GIHP and GFHT, P. Albaladejo, N. Blais, F. Bonhomme, A. Borel-Derlon, A. Cohen, J.-P. Collet, E. de Maistre, P. Fontana, D. Garrigue Huet, A. Godier, Y. Gruel, A. Godon, B. Ickx, S. Laporte, D. Lasne, J. Llau, G. Le Gal, T. Lecompte, S. Lessire, J. H. Levy, D. Longrois, S. Madi-Jebara, A. Mansour, M. Mazighi, P. Mismetti, P. E. Morange, S. Motte, F. Mullier, N. Nathan, P. Nguyen, G. Pernod, N. Rosencher, S. Roullet, P. M. Roy, S. Schlumberger, P. Sié, A. Steib, S. Susen, C. A. Tacquard, S. Testa, A. Vincentelli, P. Zufferey, A. Borel-Derlon, E. Boissier, B. Dumont, E. de Maistre, Y. Gruel, C. James, D. Lasne, T. Lecompte, P. E. Morange, P. Nguyen, V. Siguret, S. Susen

**Affiliations:** 1 Department of Hematology and Transfusion, Lille University Hospital, Lille, France; 2 Department of Hemostasis and Transfusion, CHU Lille, Lille, France; 3 Department of Anesthesia and Intensive Care, Strasbourg University Hospital, Strasbourg, France; 4 Department of Anesthesiology and Critical Care, Grenoble Alpes University Hospital, La Tronche, France; 5 Department of Anesthesiology and Critical Care Medicine, Rennes University Hospital, Rennes, France; 6 Department of Hematology Laboratory, Reims University Hospital, Reims, France; 7 Department of Anesthesia and Intensive Care, HEGP-AP-HP, Paris, France; 8 AO Istituti Ospitalieri, Cremona, Italy; 9 Duke University Hospital, Durham, NC USA; 10 Department of Hematology-Hemostasis, Tours University Hospital, CHRU Tours, Tours, France; 11 Anesthesia and Critical Care, Grenoble, France; 12 Hematology-Hemostasis, Montréal, Canada; 13 Anesthesia and Critical Care, Geneva, Switzerland; 14 Hematology-Hemostasis, Caen, France; 15 Cardiology, Paris, France; 16 Hematology-Hemostasis, Dijon, France; 17 Hematology-Hemostasis, Geneva, Switzerland; 18 Anesthesia and Critical Care, Lille, France; 19 Anesthesia and Critical Care, Paris, France; 20 Hematology-Hemostasis, Tours, France; 21 Anesthesia and Critical Care, Brussels, Belgium; 22 Clinical Pharmacology, Saint-Etienne, France; 23 Hematology-Hemostasis, Paris, France; 24 Anesthesia and Critical Care, Valencia, Spain; 25 Vascular Medicine, Ottawa, Canada; 26 Anesthesia and Critical Care, Namur, Belgium; 27 Anesthesia and Critical Care, Durham, USA; 28 Anesthesia and Critical Care, Beyrouth, Lebanon; 29 Anesthesia and Critical Care, Rennes, France; 30 Neurology, Paris, France; 31 Hematology-Hemostasis, Marseille, France; 32 Vascular Medicine, Brussels, Belgium; 33 Hematology-Hemostasis, Namur, Belgium; 34 Anesthesia and Critical Care, Limoges, France; 35 Hematology-Hemostasis, Reims, France; 36 Vascular Medicine, Grenoble, France; 37 Anesthesia and Critical Care, Bordeaux, France; 38 Emergency Medicine, Angers, France; 39 Anesthesia and Critical Care, Suresnes, France; 40 Hematology-Hemostasis, Toulouse, France; 41 Anesthesia and Critical Care, Strasbourg, France; 42 Hematology-Hemostasis, Lille, France; 43 Hematology, Cremona, Italy; 44 Cardiac Surgery, Lille, France; 45 Anesthesia and Critical Care, Saint- Etienne, France; 46 Hematology-Hemostasis, Nantes, France; 47 Hématology, Bordeaux, France; 48 Hematology-Hemostasis, Genève, Suisse

**Keywords:** COVID-19, Thrombosis, Obesity, Anticoagulant, Heparin, Coagulation

## Abstract

COVID-19 is an infection induced by the SARS-CoV-2 coronavirus, and severe forms can lead to acute respiratory distress syndrome (ARDS) requiring intensive care unit (ICU) management. Severe forms are associated with coagulation changes, mainly characterized by an increase in D-dimer and fibrinogen levels, with a higher risk of thrombosis, particularly pulmonary embolism. The impact of obesity in severe COVID-19 has also been highlighted.

In this context, standard doses of low molecular weight heparin (LMWH) may be inadequate in ICU patients, with obesity, major inflammation, and hypercoagulability. We therefore urgently developed proposals on the prevention of thromboembolism and monitoring of hemostasis in hospitalized patients with COVID-19.

Four levels of thromboembolic risk were defined according to the severity of COVID-19 reflected by oxygen requirement and treatment, the body mass index, and other risk factors. Monitoring of hemostasis (including fibrinogen and D-dimer levels) every 48 h is proposed. Standard doses of LMWH (e.g., enoxaparin 4000 IU/24 h SC) are proposed in case of intermediate thrombotic risk (BMI < 30 kg/m^2^, no other risk factors and no ARDS). In all obese patients (high thrombotic risk), adjusted prophylaxis with intermediate doses of LMWH (e.g., enoxaparin 4000 IU/12 h SC or 6000 IU/12 h SC if weight > 120 kg), or unfractionated heparin (UFH) if renal insufficiency (200 IU/kg/24 h, IV), is proposed. The thrombotic risk was defined as very high in obese patients with ARDS and added risk factors for thromboembolism, and also in case of extracorporeal membrane oxygenation (ECMO), unexplained catheter thrombosis, dialysis filter thrombosis, or marked inflammatory syndrome and/or hypercoagulability (e.g., fibrinogen > 8 g/l and/or D-dimers > 3 μg/ml). In ICU patients, it is sometimes difficult to confirm a diagnosis of thrombosis, and curative anticoagulant treatment may also be discussed on a probabilistic basis. In all these situations, therapeutic doses of LMWH, or UFH in case of renal insufficiency with monitoring of anti-Xa activity, are proposed.

In conclusion, intensification of heparin treatment should be considered in the context of COVID-19 on the basis of clinical and biological criteria of severity, especially in severely ill ventilated patients, for whom the diagnosis of pulmonary embolism cannot be easily confirmed.

## Background

COVID-19 is an infection induced by the SARS-CoV-2 coronavirus affecting mostly adults. This viral disease is mild in many patients but is characterized in symptomatic forms by an atypical interstitial inflammatory lung disease [1]. In addition, severe forms are associated with an extreme inflammatory reaction related to a “cytokine storm,” with lung involvement that can lead to acute respiratory distress syndrome (ARDS) requiring intensive care unit (ICU) management.

In critically ill patients, especially those with hypoxemia, coagulation changes reflecting inflammation are generally observed, with increased D-dimer and fibrinogen levels [2] and, more rarely, a consumptive coagulopathy associated with a poor prognosis [2].

The pathophysiology of SARS-Cov2 infection is still poorly defined, but major inflammation and hypoxemia associated with a prothrombotic state are significant features of severe forms. Chinese, Italian, North American, and French cohorts have consistently reported that severe forms affect more often elderly patients with comorbidities (hypertension, diabetes, cardiovascular or pulmonary pathology), with high mortality in those requiring ICU admission [3–6].

More recently, the impact of obesity, often associated with other comorbidities, has been highlighted in severe forms of COVID-19 [7]. A French study has also confirmed that almost half of the patients admitted to ICU are obese (with BMI > 30 kg/m^2^) and require mechanical ventilation more often [8].

A Chinese study reported frequent venous thrombotic episodes in severe COVID-19 [9], and survival was improved with heparin thromboprophylaxis [2]. In another report, venous thromboembolic events occurred in 27% of 187 Dutch patients with COVID-19 hospitalized in the ICU [10]. Further, reports from Italy, France, and Switzerland have observed frequent venous thromboembolic complications in COVID-19 with a risk that appears particularly high in patients requiring ICU admission and/or with obesity, and frequent clotting of indwelling catheters, dialysis filters, ECMO oxygenators, and arterial thrombotic events including acute limb ischemia or stroke. In addition, pulmonary embolism has recently been identified as the most common thrombotic event occurring despite thromboprophylaxis [11, 12].

However, no study has formally documented an increased thrombotic risk in COVID-19 compared to other severe infections, nor demonstrated that this risk was associated with a poor prognosis. Nevertheless, some pathophysiological features (major inflammation in particular) and the populations affected by this pathology (with comorbidities, particularly obesity) lead to further debates on the specific thromboprophylaxis treatment modalities for COVID-19.

Although low molecular weight heparin (LMWH) is the therapy of choice in patients with severe infections, current dosing strategies [13, 14] may be inadequate in patients with increased inflammatory responses and obesity and critically ill in the ICU. In addition, we are confronted with a highly challenging medical situation, with a large influx of severe patients in ICUs and in whom it is much more difficult to confirm a diagnosis of thrombosis, which explains why the implementation of curative anticoagulant treatment can sometimes be discussed on a probabilistic basis.

In this context, given the paucity of available data, the Groupe d’intérêt en Hémostase Périopératoire (GIHP) and the Groupe Français d’études sur l’Hémostase et la Thrombose (GFHT) have developed a guidance document on the prevention of thrombosis and monitoring of hemostasis in hospitalized patients with COVID-19 in order to provide support for clinical management.

These proposals, developed by a large group of reviewers, members of the GIHP and the GFHT, are organized into 4 objectives and will be modified according to the evolution of our knowledge on COVID-19.

## Practical proposals

### Objective no. 1: To define the risk of thrombosis in patients with COVID-19

#### Proposal no. 1

*All COVID-19 patients should be screened for additional thromboembolic risk factors*, especially active cancer (treatment within the last 6 months) and recent personal history (< 1 years) of thromboembolic events.

Other risk factors may be considered (age > 70 years, prolonged bed rest, postpartum, combined oral contraception).

#### Proposal no. 2

To define the thrombotic risk level according to the following:

- The severity of COVID-19 according to the requirement of supplemental oxygen (O_2_) therapy, high-flow nasal oxygen therapy, positive airway pressure support, or mechanical ventilation.

- The body mass index (BMI)

Then, 4 different levels of thromboembolic risk can be determined: low, intermediate, high, and very high (Table 1).

### Objective no. 2: Monitor the hemostasis

#### Proposal no. 3

It is suggested that the following hemostasis variables be monitored at least every 48 h: platelet count, prothrombin time (PT), activated partial thromboplastin time (APTT), fibrinogen, and D-dimer levels.

#### Proposal no. 4

In severe cases, in the event of clinical worsening, thrombocytopenia and/or a decrease in fibrinogen concentration, it is proposed that the concentration of fibrin monomers (if assay available), factors II and V, and antithrombin levels should also be measured for the diagnosis of disseminated intravascular coagulation (DIC).

#### Proposal no. 5

It is proposed that the presence of antiphospholipid antibodies should be investigated for iterative thrombosis under heparin therapy at effective doses.

### Objective no. 3: Prescribe anticoagulant therapy

#### Proposal no. 6

In all hospitalized patients, it is proposed to switch from oral anticoagulant therapy, vitamin K antagonist or direct oral anticoagulant (risk of instability and drug interactions), to curative heparin therapy.

#### Proposal no. 7

**Fig. 1:**
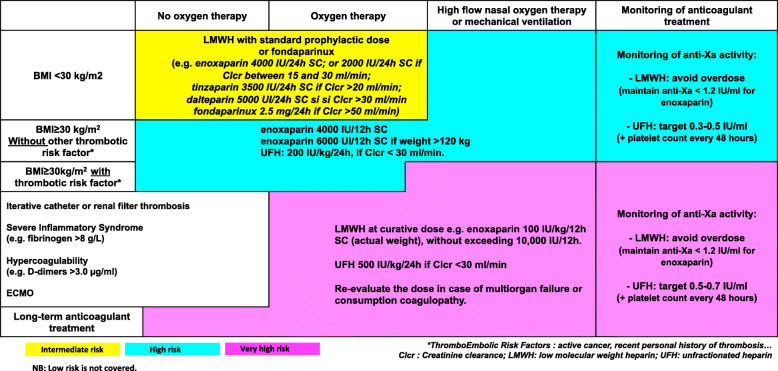
Prevention and treatment of thrombosis in hospitalized patients with COVID-19

In case of intermediate thrombotic risk (Fig. 1), it is proposed to prescribe prophylaxis with a LMWH at standard doses, e.g., enoxaparin 4000 IU/24 h SC, tinzaparin 3500 IU/24 h SC, or dalteparin 5000 IU/24 h SC. Fondaparinux 2.5 mg/24 h SC is an alternative if creatinine clearance (Clcr) is greater than 50 ml/min. In the presence of severe renal failure, an alternative to SC unfractionated heparin (UFH) can be proposed: enoxaparin 2000 IU/24 h SC for a Clcr between 15 and 30 ml/min or tinzaparin 3500 IU/24 h SC for a Clcr between 20 and 30 ml/min.

#### Proposal no. 8

In patients treated with LMWH with standard prophylactic doses, it is recommended NOT to monitor anti-Xa activity.

#### Proposal no. 9

In case of high thrombotic risk, it is proposed to prescribe an adjusted prophylaxis with intermediate doses of LMWH: enoxaparin 4000 IU/12 h SC or 6000 IU/12 h SC if weight > 120 kg. In case of renal insufficiency (Clcr < 30 ml/min), it is proposed to prescribe UFH at an initial dose of 200 IU/kg/24 h, IV.

#### Proposal no. 10

In patients treated with LMWH doses higher than the standard prophylactic regimen, it is proposed to check anti-Xa activity 4 h after the 3rd injection, then regularly in case of renal insufficiency, to monitor for levels (variable threshold value according to LMWH) due to the potential increased risk of bleeding.

#### Proposal no. 11

In case of very high thrombotic risk, it is proposed to prescribe therapeutic doses of LMWH, e.g., enoxaparin, 100 IU/kg/12 h SC, or in case of severe renal insufficiency of UFH, 500 IU/Kg/24 h, IV after a bolus of 5000 IU, and with dosage adjustment according to anti-Xa activity.

#### Proposal no. 12

In obese patients (BMI > 30 kg/m^2^) with a high or very high thrombotic risk, the following heparin doses are proposed:

- Enoxaparin 4000 IU/12 h or 6000 IU/12 h if weight > 120 kg in high-risk patients
- Enoxaparin 100 IU/kg (actual weight)/12 h SC not to exceed 10,000 IU/12 h or UFH 500 IU/kg/24 h in very high-risk patients with additional thrombotic risk factor and high-flow nasal O_2_ therapy or artificial ventilation

#### Proposal no. 13

In all patients treated with UFH, it is proposed to monitor the anti-Xa activity at least every 48 h and after each dose modification, to be maintained, if the risk of bleeding is controlled, between 0.3 and 0.5 IU/ml during reinforced prophylactic treatment (starting dose 200 IU/kg/24 h, IV) and between 0.5 and 0.7 IU/ml during therapeutic treatment (starting dose 500 IU/kg/24 h, IV, after an initial IV bolus of 5000 IU).

#### Proposal no. 14

The use of extracorporeal membrane oxygenation (ECMO) exposes the patient to a very high risk of thrombosis. In this setting, we propose therapeutic anticoagulation with UFH as soon as ECMO is initiated (independently of the ECMO flow rate), with a target anti-Xa level between 0.5 and 0.7 IU/ml.

#### Proposal no. 15

In case of an increased inflammatory syndrome (e.g., fibrinogen > 8 g/l or 800 mg/dl) and/or a rapid increase in D-dimer concentration to > 3 μg/ml (3000 ng/ml), it is suggested that the administration of therapeutic doses of heparin be considered even in the absence of clinical thrombosis, taking into account the risk of hemorrhage.

#### Proposal no. 16

For UFH, it is recommended that platelet count should be monitored at least every 48 h. A decrease in the platelet count by more than 40% between the 4th and 14th day of treatment requires a DIC assessment and a screening for heparin-induced thrombocytopenia.

#### Proposal no. 17

In the event of multiorgan failure, or a consumptive coagulopathy with decrease in fibrinogen concentration, platelet count, and factor V level, it is proposed that the dosage of heparin therapy be reassessed, as these events are associated with an increased risk of bleeding.

#### Proposal no. 18

It is proposed that the intensity and duration of thromboprophylaxis be re-evaluated according to the severity of the infection and the evolution of the resulting risk factors.

### Objective no. 4: Apply additional measures for optimal management of thrombotic risk

#### Proposal no. 19

Discontinuation of hormonal or hormone-related therapy (estrogen-progestin contraception, hormone replacement therapy, tamoxifen) is recommended in patients with COVID-19 requiring thromboprophylaxis.

#### Proposal no. 20

It is proposed to implement a specific communication pathway between the clinical units and the hemostasis laboratory, for optimal transmission of biological results (in particular, platelet count, fibrinogen, D-dimer, and anti-Xa activity) for rapid adaptation of heparin therapy.

#### Proposal no. 21

It is suggested that pulmonary embolism be investigated in any patient with sudden respiratory or hemodynamic deterioration, especially in the case of right ventricular dysfunction.

#### Proposal no. 22

A proximal vein compression ultrasonography with Doppler of the common femoral and popliteal veins is proposed in the event of any unexplained clinical worsening, or in the case of a sudden increase in D-dimer levels.

This examination may also be performed earlier in patients with a central venous catheter, especially in case of dysfunction or obstruction.

#### Proposal no. 23

The insertion of a vena cava filter is not warranted in these patients at low risk for bleeding.

#### Proposal no. 24

Intermittent pneumatic compression is an option to be considered if available in patients at a high risk of bleeding.

## Discussion

With these practical proposals, we aimed to assist clinicians in the therapy and monitoring of anticoagulation to prevent thrombosis in hospitalized patients with COVID-19. As a result, we did not address the management of a consumptive coagulopathy, which can occur in severe patients, nor certain situations such as ambulatory patients, pregnancy, or patients with underlying diseases (e.g., sickle cell disease, congenital hemostatic defects), as well as the role of thrombolysis, antiplatelet drugs, and the management of arterial thrombotic events.

We first proposed defining the level of thrombotic risk according to the severity of the disease, i.e., the presence or absence of hypoxemia and BMI (Table 1). Other additional risk factors for thromboembolism such as recent thrombosis or active cancer must also be considered.

**Table 1:** Thrombotic risk levels in patients with COVID-19 according to BMI, requirement of O_2_ or mechanical assistance, and other risk factors of thrombosis

Low risk	Intermediate risk	High risk	Very high risk
Non-hospitalized patient with BMI < 30 kg/m^2^ and no added risk factors for thromboembolism (such as active cancer, recent history of thrombosis)	BMI < 30 kg/m^2^, without the need for high-flow nasal oxygen therapy or mechanical ventilation, with or without added risk factors for thromboembolism	- BMI < 30 kg/m^2^, under high-flow nasal oxygen therapy or mechanical ventilation, with or without risk factors for thromboembolism - BMI > 30 kg/m^2^ without high-flow nasal oxygen therapy or mechanical ventilation, but with added risk factors for thromboembolism - BMI > 30 kg/m^2^ with high-flow nasal oxygen therapy or mechanical ventilation, and without added risk factors for thromboembolism	- BMI > 30 kg/m^2^ with added risk factors for thromboembolism, AND high-flow nasal oxygen therapy or mechanical ventilation - ECMO (venovenous or veno-arterial) - Unexplained catheter thrombosis - Dialysis filter thrombosis - Marked inflammatory syndrome and/or hypercoagulability (e.g., fibrinogen > 8 g/l (800 mg/dl) and/or D-dimers > 3 μg/ml or 3000 ng/ml)

Antithrombotic prophylaxis is not required in case of low risk, but is necessary in all hospitalized and immobile patients, as for any severe acute infection [13, 14], with preference given to heparins. Interestingly, a specific beneficial effect of heparin on COVID-19 is also suggested, due to its pleiotropic actions (cytokine binding, inhibition of chemotaxis, leukocyte migration, and complement activation, sequestration of inflammatory proteins) [15].

In the less severe forms and if the BMI is < 30 kg/m^2^ (intermediate risk), it is recommended, in accordance with the most recent guidelines [14], to prescribe fondaparinux or a LMWH at a standard dose (e.g., enoxaparin 4000 IU/24 h SC).

But in obese patients (BMI > 30 kg/m^2^), who present a higher thrombotic risk, we suggest increased doses of LMWH, in accordance with ESC proposals in other scenarios [16]. However, in the absence of data on the bleeding risk associated with higher doses of LMWH in the context of COVID-19, it is preferable not to initially exceed 10,000 IU/12 h of enoxaparin SC.

In ICU patients, the thrombotic risk is frequently high [17], especially in case of sepsis [18], and prophylaxis at usual doses may be ineffective, and more importantly in case of obesity [19]. Heparin therapy at therapeutic doses had been proposed during the H1N1 influenza, due to an increased thrombotic risk, particularly in patients with ARDS [20]. Moreover, the incidence of severe hemorrhagic accidents is rather low in COVID-19 patients [21, 22], whereas the thrombotic risk is high despite anticoagulation, as recently documented in 2 French cohorts [11, 12]. We therefore propose increased or even therapeutic doses (if additional thromboembolic risk factors are present) of LMWH or unfractionated heparin in COVID-19 patients hospitalized in ICUs. Thus, in obese patients with well-identified thromboembolic risk factors (active cancer or recent thrombosis in particular), therapeutic doses of LMWH adjusted according to actual weight [23] will be preferentially administered. Therapeutic dosing of heparin is also indicated in cases of thrombosis or during ECMO. However, it should also be considered in cases of a major inflammatory syndrome associated with severe pulmonary involvement, which often results in elevated fibrinogen and/or D-dimer concentrations, also indicating a severe prothrombotic state, regardless of BMI. The increase in D-dimer levels in COVID-19 is more pronounced in the most critically ill patients [24], with a concentration often greater than 3 μg/ml [2]. In addition, mortality was lower in patients whose D-dimer levels were above this threshold, when treated with heparin [25]. Therefore, these variables should be measured regularly, and a rapid increase in the level of D-dimers (outside of DIC) and/or fibrinogen should prompt to search for a thrombotic event and discuss the administration of therapeutic doses of heparin. In practice, an intensification of heparin treatment should be considered based on clinical and biological considerations of severity, but such a proposal is debated [26, 27], and clinical trials are mandatory. However in practice, when evaluation of thrombosis is not feasible, such as in critically ill ventilated patients, a probabilistic antithrombotic intensification should be considered when there is a high thrombotic risk.

Biologically, we propose to monitor prothrombin time, APTT, fibrinogen, and D-dimers at least every 48 h, combined in patients treated with UFH with monitoring of platelet count and anti-Xa activity (if curative doses and/or renal failure). This regular monitoring of hemostasis is crucial in all COVID-19 patients hospitalized with two main objectives as follows:

1. To evaluate the evolution of the disease using predictive markers of complications
2. To screen for heparin overdose, associated with a risk of bleeding, and heparin-induced thrombocytopenia (HIT) in UFH-treated patients

Anti-Xa activity, although not correlated with the occurrence of thrombosis or bleeding, especially in case of obesity [28], should be measured in patients receiving increased doses of LMWH, but is only intended to eliminate overdose (Fig. 1). Dosage adjustment to achieve a target value is therefore not recommended. In patients treated with UFH, dose adjustment based on anti-Xa activity is recommended [29]. Thus, we propose to maintain anti-Xa activity between 0.3 and 0.5 IU/ml during enhanced prophylactic treatment and between 0.5 and 0.7 IU/ml during therapeutic treatment.

Platelet count should be monitored every 48 h between days 4 and 14 of treatment with UFH to detect HIT [30]. A relative heparin resistance has been described in COVID-19 [24], but infrequently. Rarely associated with a modest decrease in antithrombin level [24], it can be resolved in most cases by increasing heparin doses. Antithrombin supplementation, which can increase the risk of bleeding in ICU patients [31, 32], is not recommended [33], as well as argatroban, which should be reserved only for patients with HIT [30, 34].

In case of unexplained and/or atypical thrombosis, antiphospholipid antibodies can also be detected [35] and could be frequent in COVID-19 [11]. However, these antibodies are often non-pathogenic in severe infections, and their persistence should therefore be checked after COVID-19 recovery.

Finally, monitoring the hemostasis of COVID-19 patients is essential for the diagnosis of DIC, which complicates severe forms [2], and should be suspected with classic manifestations including thrombocytopenia, prolongation of APTT and PT, decrease in fibrinogen levels, elevation of D-dimers, and the presence of fibrin monomers.

## Conclusion

COVID-19 is a disease with high thrombotic risk, and heparin therapy with doses of LMWH or UFH adjusted to the level of risk are required in all hospitalized patients, especially in obese and critically ill patients. Regular biological monitoring is essential to optimize anticoagulant management and treatment, and it is important to strengthen collaboration and communication between the ICUs and the hematology laboratory.

## Data Availability

Not applicable

## Abbreviations

APTT: Activated partial thromboplastin time
ARDS: Acute respiratory distress syndrome
BMI: Body mass index
DIC: Disseminated intravascular coagulation
ECMO: Extracorporeal membrane oxygenation
GFHT: Groupe Français d’études sur l’Hémostase et la Thrombose
GIHP: Groupe d’intérêt en Hémostase Périopératoire
HIT: Heparin-induced thrombocytopenia
ICU: Intensive care unit
LMWH: Low molecular weight heparin
O_2_: Oxygen
PT: Prothrombin time
UFH: Unfractionated heparin
